# Dissemination of *Orientia tsutsugamushi* and Inflammatory Responses in a Murine Model of Scrub Typhus

**DOI:** 10.1371/journal.pntd.0003064

**Published:** 2014-08-14

**Authors:** Christian A. Keller, Matthias Hauptmann, Julia Kolbaum, Mohammad Gharaibeh, Melanie Neumann, Markus Glatzel, Bernhard Fleischer

**Affiliations:** 1 Bernhard Nocht Institute for Tropical Medicine, Hamburg, Germany; 2 Mouse Pathology Core Facility, University Medical Center Hamburg-Eppendorf, Hamburg, Germany; 3 Institute of Neuropathology, University Medical Center Hamburg-Eppendorf, Hamburg, Germany; 4 Institute of Immunology, University Medical Center Hamburg-Eppendorf, Hamburg, Germany; Institut Pasteur, France

## Abstract

Central aspects in the pathogenesis of scrub typhus, an infection caused by *Orientia (O.) tsutsugamushi*, have remained obscure. Its organ and cellular tropism are poorly understood.

The purpose of this study was to analyze the kinetics of bacterial dissemination and associated inflammatory responses in infected tissues in an experimental scrub typhus mouse model, following infection with the human pathogenic strain Karp. We provide a thorough analysis of *O. tsutsugamushi* infection in inbred Balb/c mice using footpad inoculation, which is close to the natural way of infection. By a novel, highly sensitive qPCR targeting the multi copy *traD* genes, we quantitatively monitored the spread of *O. tsutsugamushi* Karp from the skin inoculation site via the regional lymph node to the internal target organs. The highest bacterial loads were measured in the lung. Using confocal imaging, we also detected *O. tsutsugamushi* at the single cell level in the lung and found a predominant macrophage rather than endothelial localization. Immunohistochemical analysis of infiltrates in lung and brain revealed differently composed lesions with specific localizations: iNOS-expressing macrophages were frequent in infiltrative parenchymal noduli, but uncommon in perivascular lesions within these organs. Quantitative analysis of the macrophage response by immunohistochemistry in liver, heart, lung and brain demonstrated an early onset of macrophage activation in the liver. Serum levels of interferon (IFN)-γ were increased during the acute infection, and we showed that IFN-γ contributed to iNOS-dependent bacterial growth control.

Our data show that upon inoculation to the skin, *O. tsutsugamushi* spreads systemically to a large number of organs and gives rise to organ-specific inflammation patterns. The findings suggest an essential role for the lung in the pathogenesis of scrub typhus. The model will allow detailed studies on host-pathogen interaction and provide further insight into the pathogenesis of *O. tsutsugamushi* infection.

## Author Summary

Many details of the pathogenesis of scrub typhus, an infection caused by the intracellular bacterium *Orientia tsutsugamushi* that is endemic in Southeast Asia, have remained unclear until today. In this study, we present an experimental self-healing mouse model of scrub typhus based on footpad skin inoculation of the human pathogenic Karp strain of *O. tsutsugamushi* that shares many features with human infection. We established a novel quantitative PCR with increased sensitivity for the measurement of bacterial organ loads of infected mice. It was thereby shown that *O. tsutsugamushi* initially accumulated in the regional lymph node and subsequently spread to many organs with the highest bacterial loads found in the lung. The predominant host cells in the lung were macrophages located in the parenchymal interstitium, rather than endothelial cells. Our data also show unexpected organ-specific differences in the dynamics of macrophage activation. This mouse model will help to advance our understanding of scrub typhus pathogenesis.

## Introduction

Scrub typhus, the human infection with *Orientia (O.) tsutsugamushi*, is a febrile, potentially lethal disease that is highly endemic in rural areas of Southeast Asia. About 1 billion people are believed to be at risk [1]. Despite detailed postmortem studies from the 1940s [2], [3] and a large number of immunological experiments conducted in the 1970s and 1980s [4], important aspects of the pathogenesis of *O. tsutsugamushi* infection have remained elusive.

To date, the dissemination kinetics of *O. tsutsugamushi* from the skin to internal organs has not been elucidated. In humans, only the eschar developing at the inoculation site is reasonably accessible for investigations of host-pathogen interactions [5]. Yet, quantitative approaches in humans assessing the involvement of internal organs are largely precluded by the invasiveness of sample retrieval, or confined to anecdotal case reports and autopsy studies. Tracking the events in the large group of subclinical or unspecific scrub typhus infections in humans, which often pass unrecognized or remain undiagnosed, will thus be challenging if not impossible. These circumstances raise the need for suitable animal models [6].

Mice belong to the wildlife host range of *O. tsutsugamushi* [7], [8]. A number of different genetic backgrounds and inoculation routes have been used to study *O. tsutsugamushi* infection in mice, including the intraperitoneal (i.p.), subcutaneous (s.c.) and intradermal (i.d.) routes [9], [10], [11], [12]. However, the mouse has so far remained an incomplete model for human infection, since no inoculation route reflects all pathogenetic details observed in humans. E.g., eschar formation is not recapitulated by any model, and i.p. infections cause a highly replicative peritonitis which primarily involves infection of peritoneal macrophages and neutrophils and may thus trigger mechanisms that are not involved in natural infection [10].

A large number of studies have analyzed *O. tsutsugamushi* infection after s.c. administration of large inocula (0.2 ml) [11], [13], [14], [15], which may equally not be representative for the natural i.d. infection by chiggers. We wanted to establish an inbred mouse model that is closer to the natural course of infection than previous models, and that would allow detailed immunological studies such as adoptive transfer of lymphocyte subsets. The skin of the footpad allows combined i.d. and s.c. inoculation [16], [17] of small volumes (up to 50 µl) and was thus chosen as injection site. This inoculation scheme was set up in analogy to the experimental mouse model of cutaneous leishmaniasis, where intradermal ear and combined footpad inoculations have largely comparable clinical outcomes [18].

In this study, we present a kinetic analysis of the clinical course in footpad-infected BALB/c mice, using the human pathogenic Karp strain of *O. tsutsugamushi*. By quantifying *O. tsutsugamushi* organ loads measured by a novel, highly sensitive qPCR, we provide the first evidence that *O. tsutsugamushi* has a distinct tropism for lung tissue in this model. Furthermore, we describe the localization and composition of histopathological changes in the lung, liver, central nervous system (CNS) and heart and demonstrate organ-specific differences in the kinetics of macrophages invasion and activation. We also provide evidence for a macrophage rather than endothelial tropism of *O. tsutsugamushi* in the lung and show that the infected cells preferentially reside in the parenchymal interstitium. Thus, this study provides essential and new insights into the pathogenesis of self-healing systemic infection with *O. tsutsugamushi*.

## Methods

### Media and bacterial culture

*O. tsutsugamushi* strains were obtained from Dr. J. Stenos (Australian Rickettsial Reference Laboratory, Geelong, Australia). Infected, γ-irradiated L929 cells were cultured in RPMI medium supplemented with 5% fetal calf serum (FCS), 2% glutamine and 2% HEPES buffer for 14 days and then passaged. Infectious inocula for *in vivo* experiments were prepared by harvesting infected cells from 14 days old cultures. Aliquots of the same stock were processed for storage in liquid nitrogen to ensure reproducibility of repeated infections. Cells were resuspended in freezing medium (45% RPMI medium, 35% FCS, 20% DMSO) and frozen in liquid nitrogen. For mock controls, non-infected L929 cells were prepared in the same way.

Methylcellulose medium contained 2/3 RPMI/5%FCS and 1/3 sterile 16.8 g methylcellulose/600 ml H_2_O.

### Immunofocus assay

Since *O. tsutsugamushi* organisms are delicate when isolated from host cells, infectious inocula for animal experiments consisted of L929 mouse fibroblasts infected with *O. tsutsugamushi* Karp. Infectivity of inocula was determined by immunofocus assay, which is based on the formation of antigen-positive foci in cell culture [19].

Briefly, 4×10^5^ irradiated L929 fibroblasts in 24-well plates were infected with diluted inocula thawed from liquid nitrogen stocks in replicates. Cell cultures were overlayed with methylcellulose medium, continued for 14 days and fixed for 2 h with 4% paraformaldehyde. Antigen-positive foci were labeled with the 2F2 monoclonal antibody (mAb) directed against the 56 kDa surface antigen of *O. tsutsugamushi* (see Methods S1 for details on mAb generation) and detected with a peroxidase-labeled anti-mouse conjugate (Dianova, Hamburg, Germany). After development of substrate, spots were counted by two independent microscopists with a standard inverted microscope. The proportion of material containing 50 spot-forming units (*sfu*) was calculated by non-linear regression of raw data in order to determine the total number of *sfu* per aliquot. Comparisons between fresh and cryopreserved inocula were not performed.

### Mice and infections

*In vivo* experiments were carried out at the animal facility of the Bernhard Nocht Institute for Tropical Medicine in Hamburg with the permission of the Health Authorities of the State of Hamburg, Germany. Female 6–7 week-old BALB/c mice were purchased from Charles River (Sulzfeld, Germany). Mice were kept in individually ventilated cages within BSL-3 facilities. 8–10 weeks old mice received, unless otherwise stated, a 50 µl inoculum containing 5–10×10^3^ *sfu* of *O. tsutsugamushi* Karp or uninfected irradiated L929 cells in the right hind foot pad. The course of infection was followed by assessing symptom severity by a clinical score (Fig. S1).

### Quantitative real time PCR

Organ samples were homogenized in 200 µl PBS, using Precellys ceramic beads (1.4/2.8 mm) in a Precellys homogenizer (Peqlab, Erlangen, Germany). DNA extractions from suspensions were performed using the QiaAmp DNA mini kit (Qiagen, Hilden, Germany) following the manufacturer's instructions. DNA concentrations of the extracts were determined with a Nanodrop photometer (Thermo Scientific, Wilmington, USA) and adjusted to 5 or 10 ng/µl for normalization to total tissue DNA content. Organ loads or loads of intracellular bacteria were determined by qPCR for the multi copy conjugative transfer protein D (*traD*) genes (*traD*-fw: 5′-CACAACATCCAAATGTTCAG-3′; *traD*-rv: 5′-GCACCATTCTTGACGAAA-3′) in a SYBR green qPCR on a Roche LightCycler 480 II instrument. In a total volume of 10 µl, the PCR reaction mix contained a final concentration of 600 nM of each primer (Tibmolbiol, Berlin, Germany), 200 µM dNTPs, 100 µg/mL bovine serum albumin (BSA), SYBR green (Invitrogen, Darmstadt, Germany) and 5 U/µL Hotstar taq DNA polymerase (Qiagen, Hilden, Germany) as well as 10 ng (blood or tissue culture samples) or 20 ng (organ samples) of template DNA. Enzyme activation at 95°C for 15 min was followed by amplification in 45 cycles of 10 s at 94°C, 15 s at 58°C and 20 s at 72°C. Each sample was measured in duplicates. Specificity of the product was confirmed by melting curve analysis.

Absolute quantification of *traD* copy numbers in a given sample was performed by the 2nd derivative maximum method with reference to a linearized plasmid standard. Results were depicted as log values of *traD* copy numbers. For calculation of genome equivalent numbers, a qPCR specific for the 56 kDa protein of *O. tsutsugamushi* [20] was adapted to the 2-step SYBR green format. For quantification by the 16s rRNA gene, another previously published qPCR was used [21].

### Probit analysis

A DNA extract with a known *O. tsutsugamushi* genome copy number was diluted serially in half-logarithmic (10^0.5^-fold) dilutions, and 8–16 replicates of each dilution were analyzed by qPCR. The 95% detection limit (LOD95) was calculated using the PriProbit software [22].

### Blood biochemistry, histology and immunofluorescence

Serum AST (aspartate aminotransferase) and ALT (alanine aminotransferase) activities were measured by using commercially available colorimetric assays (Reflotron, Roche Diagnostics, Mannheim, Germany).

For histology and immunohistochemistry (IHC) stains, standard methods were used. For immunofluorescence imaging, lung tissue was fixed with 4% paraformaldehyde overnight and frozen in cryopreservation medium (TissueTek O.C.T Compound, Sakura Finetek, Torrance, USA). Samples were sequentially reacted with goat-anti-CD31 (BD Bioscience, Heidelberg, Germany) or rabbit-anti-IBA1 (ionized calcium binding adapter molecule 1; Wako, Neuss, Germany) overnight; AlexaFluor488 donkey-anti-rabbit or AlexaFluor488 donkey-anti-goat; 2F2 mAb; AlexaFluor594 donkey-anti-mouse (Life Technologies, Darmstadt, Germany). DAPI (4′,6-diamidino-2-phenylindole; Sigma, Germany) was used for nucleus counterstains (see supplementary material for details). Sections were embedded in Fluoromount G (Southern Biotech, Birmingham, USA) and viewed with a BZ-9000 Keyence fluorescence microscope or an Olympus Confocal Microscope.

### ELISA

Serum samples were diluted 1∶10 in 0.1% BSA/PBS and measured by a standard sandwich ELISA for the presence of interferon (IFN)-γ (R&D Systems, Wiesbaden, Germany), according to the manufacturer's instructions.

### Macrophage cell culture

An immortalized mouse macrophage cell line was kindly provided by Douglas Golenbock, University of Massachusetts Medical School, Worcester MA, USA [23]. *O. tsutsugamushi* Karp was purified from infected L929 cells 2–3 weeks post infection (p.i.) by disruption with sterile glass beads (1 mm diameter), by rocking for 5 min at 1,400 rpm on a horizontal shaker at room temperature. To remove cell debris, the suspension was centrifuged for 1 h at 1,200 rpm. The supernatant containing purified bacteria was pelleted at 4,000 rpm for 30 min and resuspended in the required volume. In 24-well plates, 2×10^5^ macrophages were infected with purified *O. tsutsugamushi*. Recombinant IFN-γ (100 IU; Millipore, Billerica, USA), the inducible nitric oxide synthase (iNOS) inhibitor N-monomethylarginine (NMMA, 1 mM; Sigma, Germany) or the anti-IFN-γ mAb XMG1.2 (1 µg/ml) were added to the culture. After 3 days, DNA was extracted from detached macrophages, and the bacterial load was quantified by *traD* qPCR.

### Statistical analysis

Data were analyzed using the Graphpad Prism 5.0 software. Descriptive statistics show mean ± SD. Hypotheses were tested by two-tailed t test, or by one-way or two-way analysis of variance (ANOVA) with Bonferroni post correction. A p value of ≤0.05 was considered significant. For quantification of stock infectivity, one-phase exponential association curves were calculated.

### Ethics statement

The animal protocol was reviewed and approved by the Animal Protection Commission and the Health Department of the State of Hamburg, Germany (approval number 74/09). The animal protocol adheres to the national guidelines as regulated by the German Animal Welfare Act.

## Results

### Development of a highly sensitive qPCR for quantification of *O. tsutsugamushi*

One of the major restraints in experimental scrub typhus research has been the exactness of pathogen quantification, which has relied on light microscopy in the past [10]. For qPCR-based quantification of *O. tsutsugamushi*, the majority of methods are based on the quantification of single copy genes that often yield high cycle threshold (Ct) values and low bacterial copy numbers, thus compromising statistical analyses [9], [20], [24], [25].

In order to increase qPCR robustness especially for low bacterial concentrations, we designed a qPCR based on the amplification of multiple alleles of the *traD* (conjugative transfer protein D) gene (e.g. NCBI Gene ID 6336199) which encode for a subunit of type IV secretion systems [26]. Primer analysis by Primer-BLAST (http://www.ncbi.nlm.nih.gov/tools/primer-blast/) yielded no relevant unspecific amplification products. Negative amplification results were obtained from 27/27 non-rickettsial bacterial and fungal strains (Table S1). Occasional weak amplification was obtained from some rickettsial strains by *traD*, but not by 16s or 56 kDa qPCR [20], [21], suggesting that very high concentrations of rickettsial DNA might interfere with *traD* quantification of *O. tsutsugamushi*. For the purpose of experimental quantification, this specificity was satisfactory, since no cross-reactions were found with commensals, and co-infections with other rickettsiae were not part of this study.

**Figure 1:**
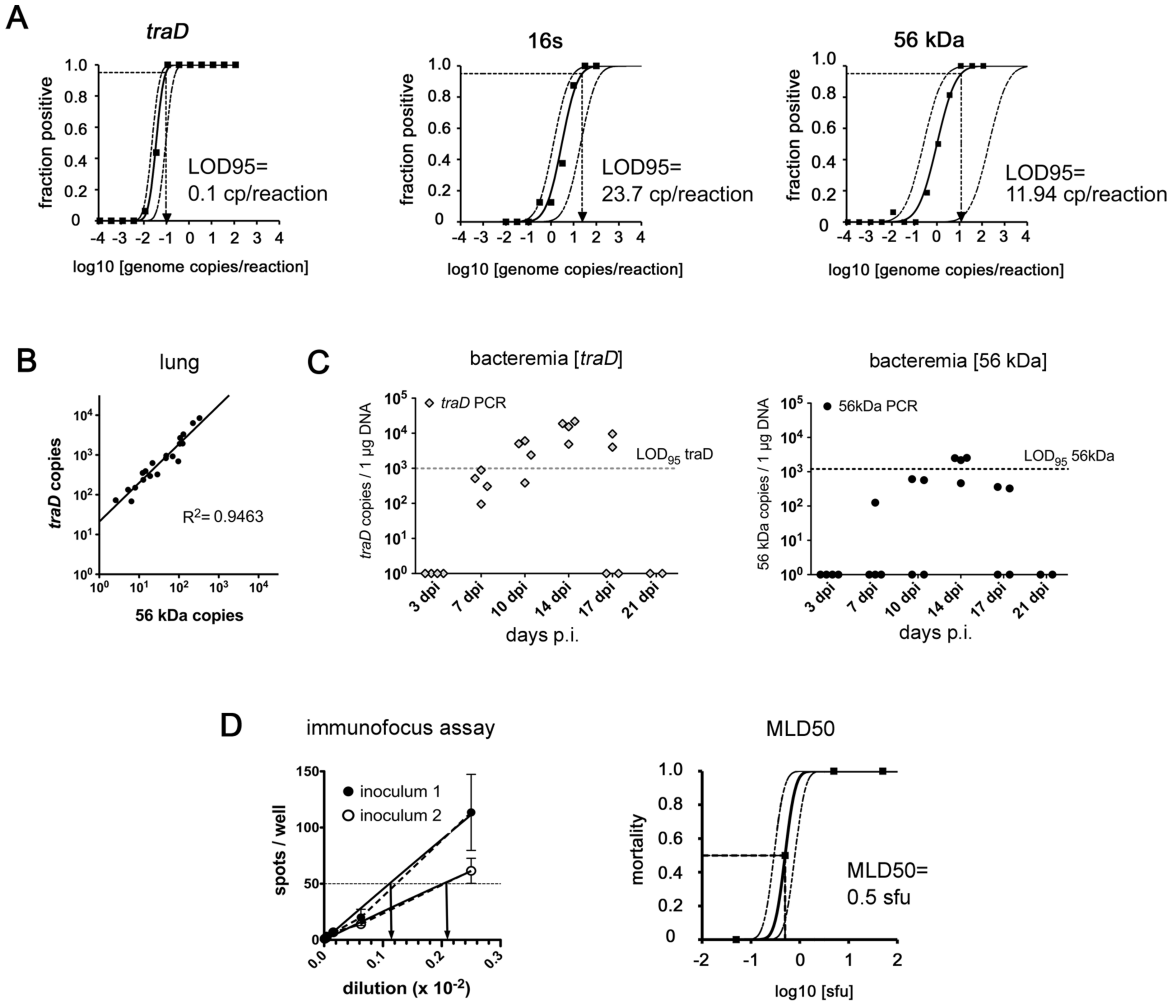
Analytical sensitivity of *O. tsutsugamushi traD* qPCR. A, Genomic DNA of *O. tsutsugamushi* was extracted from L929 cell cultures infected with *O. tsutsugamushi* Karp. Half-logarithmic dilutions were prepared. Eight to sixteen replicates of each dilution (shown on the *x*-axis) were measured for the presence or absence of *O. tsutsugamushi* by multi copy *traD* qPCR (left panel), single copy 16s (middle panel) and single copy 56 kDa qPCRs (right panel). The fraction of positive results is shown on the *y*-axis. Data were processed for probit analysis to determine the LOD95, shown on each panel. B, Balb/c mice were footpad-infected with *O. tsutsugamushi* Karp. Lung samples were retrieved between day 7 and day 21 p.i. and processed for DNA extraction. From every sample, the copy numbers of *traD* genes and 56 kDa genes were measured by qPCR. The graph shows a tight correlation between both methods. C, Blood samples from *O. tsutsugamushi*-infected Balb/c mice were quantified using *traD* (left) or 56 kDa qPCR (right) and plotted in graphs depicting the gene-specific LOD95. D, Two *O. tsutsugamushi* stocks with different infectivity were quantified by immunofocus assay (left panel). From four different inoculum dilutions, the number of spots per well was counted (dotted line: connecting line between means). To compare infectivity of the two inocula, the hypothetical inoculum dilution yielding 50 *sfu* (indicated by arrows), a medium value in the well-readable part of the graph, was calculated by non-linear regression (solid line). To correlate the concentration obtained by immunofocus assay to infectivity *in vivo*, Balb/c mice were infected with tenfold dilutions of a quantified *O. tsutsugamushi* inoculum (n = 4 per group). Within a time period of 42 days, mortality of the inoculum was assessed, and the MLD50 was determined by probit analysis (right panel).

The sensitivity of our *traD* qPCR was compared with two single copy qPCRs targeting the genes for 56 kDa and 16S rRNA [21]. Replicate testing of DNA extracts from *O. tsutsugamushi* Karp-infected cell cultures was performed in all three assays to determine the 95% limits of detection (LOD95) by probit analysis [27]. The single copy qPCRs had detection limits >10 genome copies/reaction (16s rRNA qPCR: 23.7 genome copies/reaction; 56 kDa qPCR: 11.9 genome copies/reaction; Fig. 1A). In contrast, the *traD* qPCR yielded a LOD95 of only 0.1 genome copies/reaction. Thus, in comparison to single copy qPCRs, our novel *traD* multi copy qPCR increased the sensitivity of *O. tsutsugamushi* Karp detection by more than 100-fold.

No prediction regarding the specificity of our *traD* primers for other strains of *O. tsutsugamushi* was possible due to lack of genomic data. Replicate testing and probit analysis was therefore performed for the other three *O. tsutsugamushi* strains Kato, Gilliam-like and Sido, by comparing *traD* and 16s qPCRs. Depending on the strain, the *traD* qPCR lowered the LOD95 by about 30- to 70-fold (Fig. S2). Thus, quantification by *traD* qPCR highly increased the sensitivity of *O. tsutsugamushi* detection, as shown in four genetically different strains.

It was then analyzed whether quantification by *traD* allows inferences about the number of bacterial copies in tissue samples. Lung samples from infected Balb/c mice collected at 7–21 days p.i. were quantified by both *traD* and 56 kDa qPCRs. Fig. 1B shows a high degree of correlation (R^2^ = 0.9463) between both quantification methods. Quantification by *traD* qPCR is thus a valid surrogate measure for bacterial copy numbers at both high and low bacterial concentrations. Furthermore, quantification of bacteremia in footpad-infected Balb/c mice by *traD* and 56 kDa qPCR at different time points showed that more blood samples were tested positive by *traD* compared to 56 kDa qPCR. Also, more samples yielded results above the LOD95 (Fig. 1C). This shows that the use of a multi copy qPCR is warranted to avoid false-negative results. Lower variations in repetitive testing also help to reduce the number of mice needed for statistical analysis. The *traD* qPCR was therefore used for all *O. tsutsugamushi* quantifications in this study, unless otherwise indicated.

### Inocula can be quantified by immunofocus assay

For standardization of *in vivo* experiments, *O. tsutsugamushi* inocula have usually been quantified by mouse lethal dose 50 (MLD50) or murine infectious dose 50 measurements [9], [28]. Since repeated quantification of different lots of cryopreserved inocula by *in vivo* testing is ethically questionable, we developed an *in vitro* immunofocus assay for *O. tsutsugamushi*. The principle is based on immunolabeling of infected foci in methylcellulose-overlayed cell cultures (24-well format) and has proven useful for various viral pathogens [19]; one example is shown in Fig. 1D (left panel).

We wanted to know how the immunofocus assay dose related to the i.p. MLD50. Thus, Balb/c mice were infected with diluted doses of an immunofocus assay-quantified inoculum in order to determine the MLD50 by probit analysis as reported previously [29] (Fig. 1D, right panel). 1 MLD50 corresponded to 0.5 *sfu*. Thus, quantification of infectious units in the inoculum was similar in both methods; the immunofocus assay may thus replace MLD50 testing. A quantification of the number of single infectious *O. tsutsugamushi* organisms per 1 *sfu* was not attempted.

### Development and resolution of clinical signs during *O. tsutsugamushi* infection

The human pathogenic *O. tsutsugamushi* Karp strain and inbred Balb/c mice were used for the establishment of our scrub typhus mouse model. When s.c. infected, Balb/c mice mount a protective immune response to otherwise lethal i.p. Karp infections [11] and thus represent an excellent model to study the development of protective immunity in scrub typhus.

The skin of the footpad allows combined i.d. and s.c. inoculation [16], [17] of volumes up to 50 µl, thus approximating the natural infection route more closely than formerly used s.c. models. An infection dose of 5,000 *sfu* of *O. tsutsugamushi* Karp was chosen in preliminary studies, since this dose produced clear symptoms of systemic infection between 2–3 weeks p.i.

**Figure 2:**
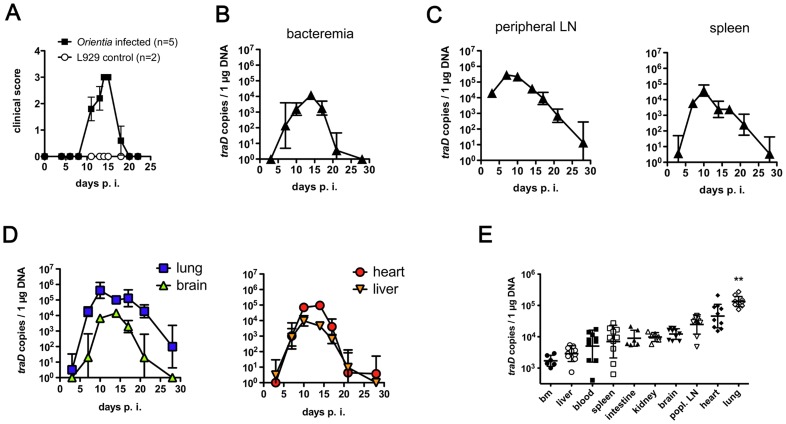
Time course of symptoms and bacterial loads in organs of *O. tsutsugamushi*-infected mice. BALB/c mice received an inoculum containing 5×10^3^ *sfu* of *O. tsutsugamushi* in the hind footpad. A, The course of infection was monitored by assessing the clinical score. B–D, At 3, 7, 10, 14, 17, 21 and 28 days p.i., mice were sacrificed, and loads of *O. tsutsugamushi* in DNA extracts of blood or indicated organs were determined by *traD* qPCR (*n* = 4). B, Bacteremia. C, Secondary lymphoid organs (peripheral lymph node (LN), spleen). D, Lung, brain, heart and liver. Graphs show combined results of two independent experiments (n = 4; mean ± SD). E, Organ loads on day 14 p.i. Bm, bone marrow (n = 6–10, mean ± SD. Data were pooled from 2–3 independent experiments. ** p<0.01 comparing lung to any other group, as determined by unpaired two-tailed *t* test).

To describe the course of symptoms in mice footpad-infected with *O. tsutsugamushi*, we created a graded scoring system that has not been described previously (Fig. S1). Uninfected L929 fibroblasts were used as mock control. As shown in Fig. 2A, footpad-infected BALB/c mice started to develop symptoms after day 14, and recovered by day 21 p.i. Mock-infected controls did not show any symptoms. Thus, an acute disease phase of about 10 days was found in our model. Skin lesions did not develop at the primary infection site. It was not investigated whether other *O. tsutsugamushi* strains elicit similar symptoms.

### Quantification of *O. tsutsugamushi* by qPCR in different tissues of infected mice

*O. tsutsugamushi* has been found in many organs of infected patients and experimental animals, including lung, heart, brain, liver, spleen, kidney, pancreas, appendix and skin [9], [30], [31], [32]. A clear organ preference of *O. tsutsugamushi* has never been revealed, and the route and kinetics of dissemination are unknown. In order to track the spread of *O. tsutsugamushi*, we measured the bacterial load in blood as well as secondary lymphatic and parenchymal organs every 3–4 days by *traD* qPCR.

*O. tsutsugamushi* appeared in blood at day 7 p.i., and bacteremia peaked at day 14 p.i. (Fig. 2B). Blood samples were negative on day 28 p.i. In the draining lymph node, *O. tsutsugamushi* was detectable at high levels at day 3 p.i., peaked at 7 days p.i. and steadily declined afterwards (Fig. 2C). Maximum spleen loads were reached at day 10 p.i.

Pneumonia, hepatitis, myocarditis and meningoencephalitis are common manifestations of solid organ involvement in scrub typhus. We therefore analyzed the dynamics of bacterial loads in lung, liver, heart and brain. The highest loads were found after day 10 in the lung, about 50-fold higher than in the brain (Fig. 2D, left panel). The organ affected with the second-highest load was the heart, where the peak was between 10 and 14 days p.i. At day 14 p.i., the heart load was still 7-fold higher compared to the liver (Fig. 2D, right panel).

Since bacteremia was maximal at day 14 p.i., the bacterial loads of ten different organs retrieved at that time point were compared (Fig. 2E). *O. tsutsugamushi* was detectable in all organs, including intestine and bone marrow. Interestingly, heart and lung had surpassed the bacterial load of the popliteal lymph node, while all others remained below. At 14 days p.i., lung loads were significantly higher compared to any other organ.

The results show that after footpad infection, *O. tsutsugamushi* initially accumulated in the regional lymph node. *O. tsutsugamushi* then spread to internal organs, but had a prominent tropism for lung and heart tissue, while lower bacterial loads were found in liver and brain.

### Self-limiting hepatitis during acute *O. tsutsugamushi* infection

**Figure 3:**
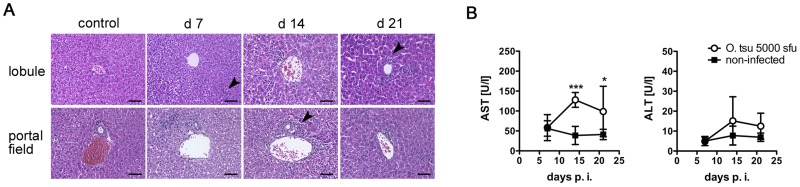
Liver pathology during *O. tsutsugamushi* infection. BALB/c mice received an inoculum containing 5×10^3^ *sfu* of *O. tsutsugamushi* in the hind footpad, or were mock-infected. A, Representative details from HE-stained liver sections show the development of hepatic inflammation. D 7, predominant inflammation of the distal parts of the lobule (arrowhead), d 14: panlobular inflammation; d21: predominant centrilobular inflammation (arrowhead). Scale bars (A–C) 50 µm. B, AST (left panel) and ALT (right panel) activities during the course of infection were quantified from serum samples taken on d 7, 14 and 21 p.i. Data from two independent experiments are shown as mean ± SD (n = 7); *, p<0.05; ***, p<0.001 by two-way ANOVA with Bonferroni's post correction.

Following the quantification of bacterial loads of infected organs, the histopathological changes during infection were analyzed. Liver involvement is a common feature in acute scrub typhus [32], [33], [34], [35]. Fig. 3A shows that early in infection, inflammation mainly affected the periphery of the hepatic lobule, while after 14 days p.i., the liver showed panlobular inflammation (Fig. 3A, upper row, arrowheads). At 21 days p.i., inflammation had begun to resolve, and mainly centrilobular foci of infiltrating cells were seen. Inflammation of portal fields started at day 7 and was maximal at day 14 p.i. (Fig. 3A, lower row; arrowhead).

Lesions in the centrilobular areas developing between day 14 and 21 p.i. were associated with a collapse of the reticular fiber network (Fig. S3, left panels; arrowheads), a sign for hepatocyte destruction. The periportal inflammations, which appeared earlier in infection (Fig. 3A), were also accompanied with a collapse of reticular fibers (Fig. S3, right panels; arrowhead).

Elevations of liver transaminases in serum showed kinetics similar to the cellular infiltration:

While on day 7 p.i. no changes were seen, serum AST activity at day 14 p.i. increased by 3–4 fold. AST activity was still elevated on day 21 p.i. (Fig. 3B, left panel). In contrast, serum ALT levels did not change significantly during infection (Fig. 3B, right panel).

Our findings show that *O. tsutsugamushi* infection in the footpad Balb/c mouse model causes mild, self-limiting hepatitis. Since transaminase levels peaked together with the maximum of cellular infiltration, and areas of hepatic inflammation coincided with hepatocyte loss, an immunopathological mechanism may be the cause of scrub typhus hepatitis.

### Scrub typhus meningoencephalitis: Glial activation and T cell infiltration

**Figure 4:**
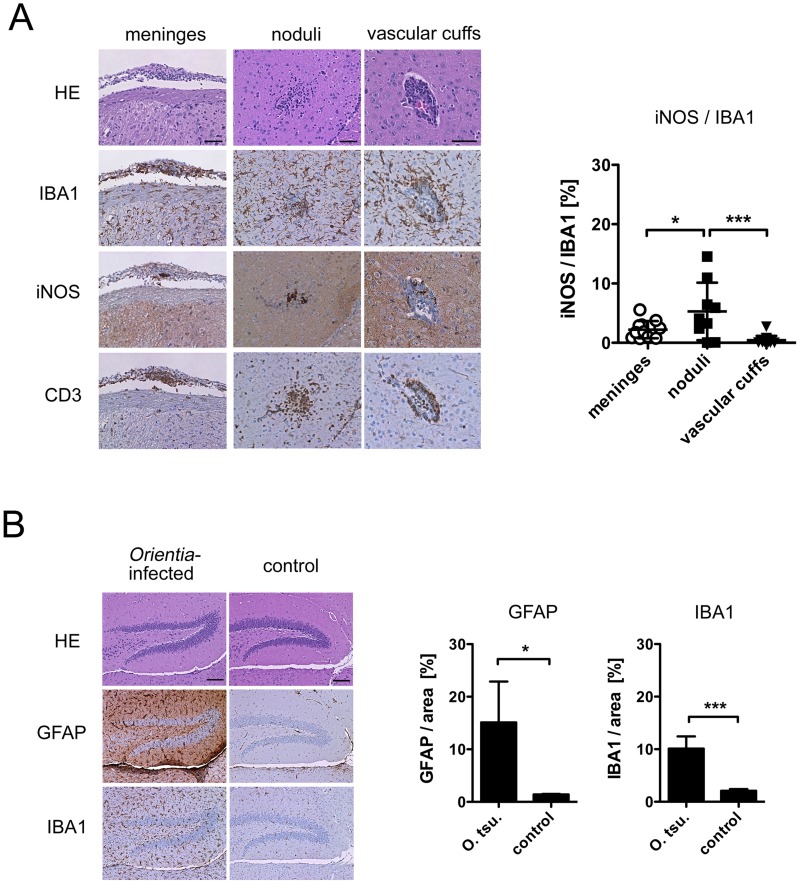
Histopathological evidence of meningitis and encephalitis. Balb/c mice received an inoculum containing 5×10^3^ *sfu* of *O. tsutsugamushi* or a mock inoculum in the hind footpad. Serial sections from brain samples (d 21 p.i.) were processed for histopathological analysis. A, Representative meningeal (arachnoidea, left panels) and parenchymal lesions (thalamus, middle and right panels) in HE stain and IHC for IBA1, iNOS and CD3. Parenchymal lesions consisted of nodular structures (middle) or perivascular cuffs (right). The iNOS/IBA1 ratio in the three lesion sites was quantified (n = 11–13 lesion sites from 2 animals) and depicted as mean ± SD; * p<0.05; *** p<0.001 by two-tailed t test. B, Serial sections of hippocampus stained by HE and IHC for GFAP (astrocytes) and IBA1 (microglia/macrophages) reveal increased glial abundance in infected brains (left panels) compared to control samples (right panels). Quantitative data are depicted as mean ± SD, * p<0.05; *** p<0.001 by two-tailed t test (n = 4 hippocampus areas from 2 animals). Scale bars: (A) 50 µm; (B) 100 µm.

Meningitis or meningoencephalitis are common complications of scrub typhus in humans [36], [37]. Whole brain samples from BALB/c mice footpad-infected with *O. tsutsugamushi* collected at 7, 14 and 21 days p.i. were processed for histopathology. Inflammatory alterations discernible in HE stains appeared at day 21 p.i. Lesions were found in the meninges and the brain parenchyma, e.g. in the thalamic region (Fig. 4A).

We analyzed the cellular composition of these lesions with IBA1, iNOS and CD3 stains (Fig. 4A). In the CNS, IBA1 was originally thought to specifically stain microglial cells, especially activated microglia [38], [39], but later studies showed that blood-derived macrophages equally stain positive for this marker under inflammatory conditions [40]. IBA1 does therefore not differentiate between both cell types and should thus be regarded as a marker for all professional phagocytic cells in the CNS.

The meninges were thickened and showed infiltrations of CD3-positive T cells and IBA1-positive cells. In the parenchyma, two different lesion patterns were seen: nodular structures with actual parenchymal infiltration of T cells (Fig. 4A, middle panels), and tight vascular cuffs associated with larger gauge blood vessels (Fig. 4A, right panels). Interestingly, the highest content of iNOS-positive cells was seen in the parenchymal noduli. Only isolated B cells, and no neutrophils or astrocytes contributed to the formation of these lesions (data not shown). Increased abundance of glial cells was observed for both microglia and GFAP (glial fibrillary acidic protein)-positive astroglia at day 21 p.i. (Fig. 4B).

Thus, activation of glial cells in the brain parenchyma, with contribution of both astrocytes and microglia/macrophages, as well as infiltration of T cells with a perivascular predominance were the hallmarks of CNS inflammation during *O. tsutsugamushi* infection. The nodular infiltrative lesions had a higher content of activated iNOS-expressing cells compared to meninges and vascular cuffs.

### Three patterns of pulmonary tissue lesions in acute scrub typhus

Since *O. tsutsugamushi* preferentially infected the lung, we also investigated the development of pulmonary pathology. Whole lung samples from BALB/c mice footpad-infected with *O. tsutsugamushi* were taken for histopathological examination 7, 14 and 21 days p.i.

**Figure 5:**
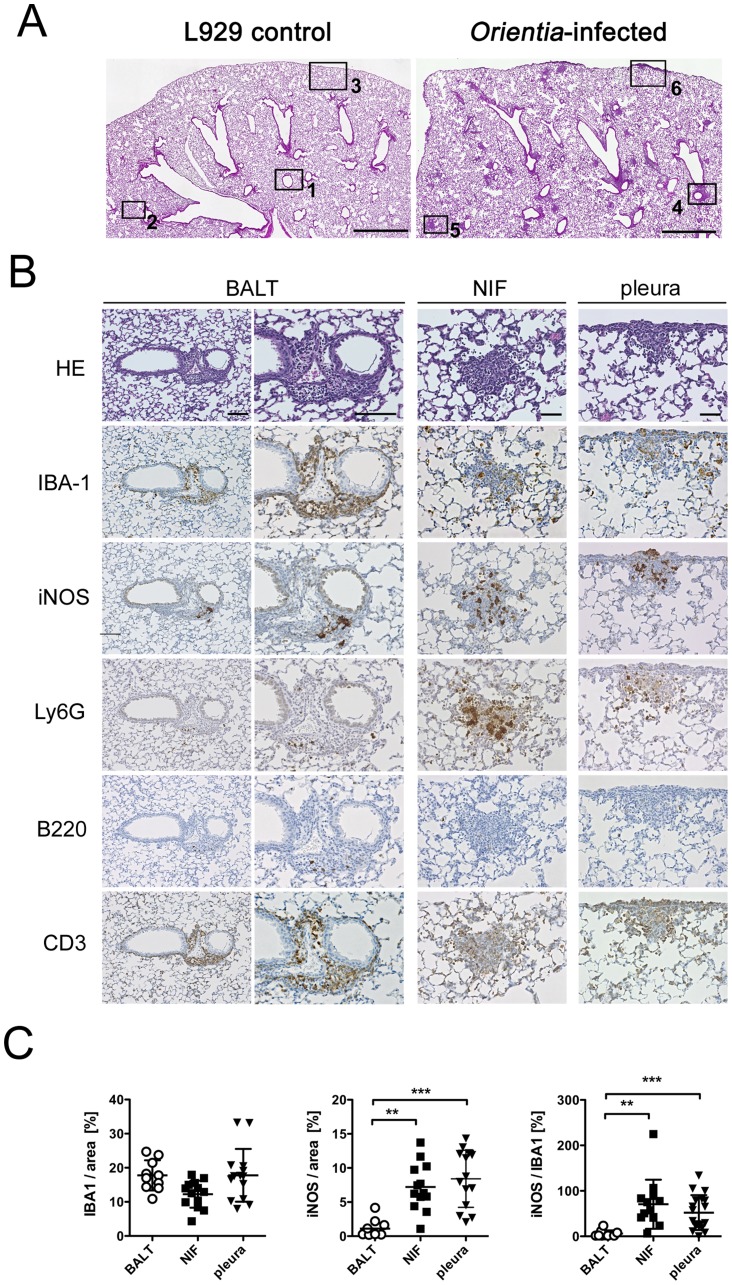
Pulmonary tissue lesions caused by *O. tsutsugamushi*. Balb/c mice were infected with 5×10^3^ *sfu* of *O. tsutsugamushi* or mock-infected with L929 cells via the hind footpad. A, Representative H&E-stained sections from lungs, taken on day 21 p.i. Upper panels: Overview of controls (left) and *O. tsutsugamushi*-infected (right) lungs, with rectangles specifying specific lesion areas (scale bar = 1 mm). B, Serial sections of lungs from *O. tsutsugamushi*-infected Balb/c mice (day 21 p.i.) were stained by HE and by immunohistochemistry for macrophages (IBA1), iNOS, neutrophils (Ly6G), B cells (B220) and T cells (CD3). Corresponding areas of the three characteristic localizations of infiltrates are shown (BALT, NIF, pleura). Scale bars: BALT, 100 µm; NIF and pleura, 50 µm. C, The IBA1- and iNOS-positive areas were quantified in BALT, NIF and pleura lesions and expressed in % of the total area and as iNOS/IBA1 ratio. Data are depicted as mean ± SD, ** p<0.01; *** p<0.001 by two-tailed t test (n = 10–14 areas from 2 animals).

During the first two weeks after infection, hematoxylin/eosin (H&E)-stained sections did not show conspicuous alterations. At day 21 p.i. the infected animals developed inflammatory infiltrates, while L929-mock-treated animals showed no pathological changes (Fig. 5A, details 1–3). The cellular infiltrates were typically found in peribronchial areas, in the parenchyma and in the visceral pleura. Peribronchial lesions showed orientation towards the adjacent arterial blood vessels (Fig. 5A, detail 4), the characteristic localization of inducible bronchus-associated lymphatic tissue (BALT). Lesions in the alveoli had a nodular appearance (Fig. 5A, detail 5). The visceral pleura was focally invaded by inflammatory infiltrates (Fig. 5A, detail 6).

For a detailed analysis of the cellular composition, pulmonary serial sections were stained for macrophages, iNOS, neutrophils, B cells and T cells. As shown in Fig. 5B (left panels), macrophages formed a unicellular layer around bronchi and, together with T cells, were the predominant cell type in BALT lesions. Some neutrophils were found in these areas, and B cells appeared focally but in small numbers. Although these structures had the typical localization of BALT, they contained only very small B cell areas, unlike the larger germinal centers found in BALT caused by other pulmonary infections [41], [42]. The nodular alveolar lesions contained macrophages, but also neutrophils and T cells (Fig. 5B, middle panels). Notably, Ly6G-stained cells appeared relatively large and fuzzy in these regions. B cells were barely detected. According to their localization, these lesions correspond to nodular inflammatory foci (NIFs), similar to lesions described in cytomegalovirus-infected lungs [43]. The lesions in the pleura had a similar cellular composition (Fig. 5B, right panels).

Quantification of IBA1-positive cells showed that all three lesion sites had a similar macrophage content (Fig. 5C). However, significantly more iNOS-positive cells and a higher iNOS-/IBA1 ratio were found in NIF and pleura lesions, compared to the perivascular BALT (Fig. 5C). This observation parallels our findings in the brain, where the infiltrative parenchymal lesions also had a higher content of iNOS-expressing cells (Fig. 4A). This suggests that activation of macrophages may be differently regulated in perivascular and infiltrative inflammatory sites, resulting in stronger iNOS induction in the latter. Possibly, even cells other than macrophages may become activated and express iNOS in these regions (Fig. 5C, right panel).

In summary, it was found that the peak of pulmonary invasion by *O. tsutsugamushi* was followed by pleural, parenchymal and peribronchial infiltrations. Perivascular orientation was a typical finding. The peribronchial/perivascular BALT lesions consisted of T cells and iNOS-negative macrophages, while NIFs in the lung parenchyma and pleural lesions harbored significantly more iNOS-positive macrophages, as well as neutrophils and few B or T cells.

### Intact and degraded bacteria are found in regions of developing pulmonary inflammation

We assumed that at the time of maximal *O. tsutsugamushi* DNA load, intact bacteria were still present within lung tissue. To demonstrate their localization, fixed cryosections of lung samples from day 14 p.i. were examined by immunohistochemistry, using the 2F2 mAb and co-staining for the endothelial marker CD31 or IBA1 in order to differentiate between an endothelial or macrophage tropism.

**Figure 6:**
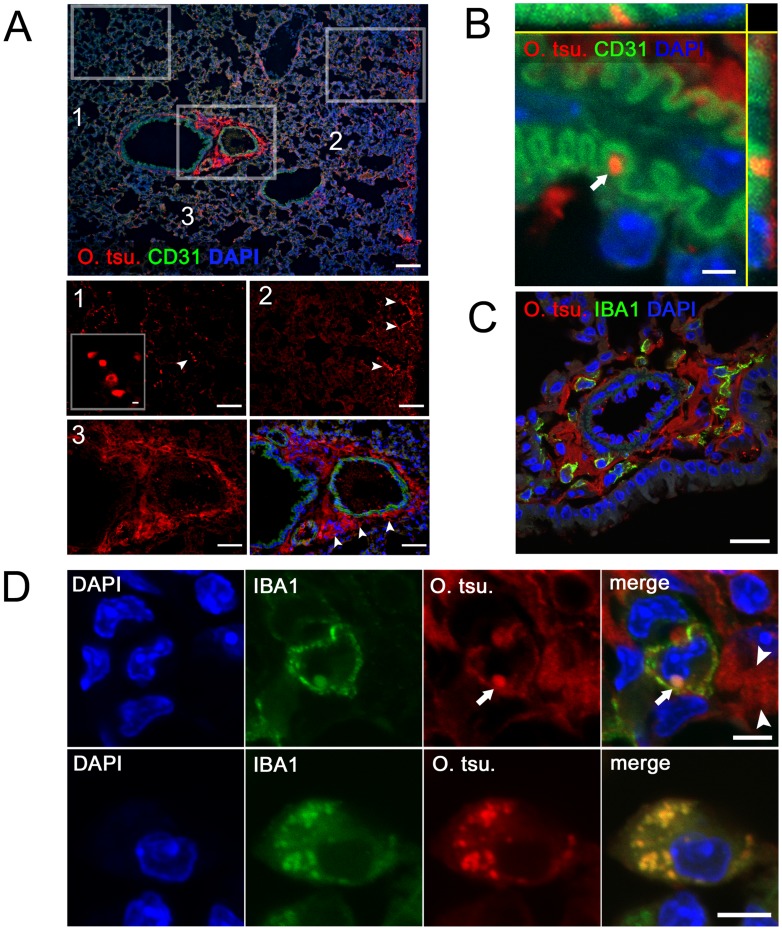
Localization of *O. tsutsugamushi* by immunohistochemistry in the lung. Balb/c mice were infected with 5×10^3^ *sfu* of *O. tsutsugamushi* via the hind footpad. A, Frozen sections of paraformaldehyde-fixed lung specimens from day 14 p.i. were stained for *O. tsutsugamushi* (2F2 mAb, red) and endothelial cells (CD31, green), and counterstained with DAPI (blue). Lung tissue imaged by epifluorescence microscopy. Rectangular insets indicate areas of higher magnification. 1, Detail of parenchyma; 2, detail of pleura; 3, detail of peribronchial/perivascular tissue (BALT). Arrowheads indicate the presence of 56 kDa antigen. Scale bars: overview, 100 µm; details, 50 µm; detail 1 (inset), 3 µm. B, endothelium-associated bacterium (arrow) imaged by confocal microscopy and maximum projection of partially resliced z-stack. Scale Bar: 2 µm. C, Extracellular antigen is abundant in perivascular BALT. Scale Bar: 20 µm. D, Infected macrophage from BALT with both surface and intracellular IBA1 expression. Arrow points to an intracellular bacterium; arrowheads indicate extracellular antigen depositions. Lower panels: Highly infected macrophage located in lung parenchyma, with intracellular IBA1 expression that co-localizes with *O. tsutsugamushi*. Scale Bars: 5 µm.

As shown in Fig. 6A, *O. tsutsugamushi* antigen was found scattered in spatially separated compartments of the lung (Fig. 6A, details 1–3). Three typical localizations were identified. Intact bacteria with coccoid shape, in some cases with a clear distinction of the bacterial cell wall, were demonstrable in lung parenchyma (Fig. 6A, detail 1). The largest accumulation of antigen was found in the pleura and perivascular BALT (Fig. 6A details 2 and 3). These structures consisted mostly of fuzzy aggregates of bacterial antigen, suggesting the presence of fragmented or degraded bacterial remnants.

Despite a close spatial relationship between bacteria and CD31-positive blood vessels, confocal imaging revealed that bacteria were merely in very close contact to endothelial cells. They protruded from infected cells crossing the endothelium, but were not located intracellularly (Fig. 6B). True intraendothelial infection was not found at any instance.

In perivascular BALT (Fig. 6C) and pleura, the majority of structures stained by the 2F2 mAb were extracellular. In BALT areas, only a small number of IBA1-positive cells harbored singular intracellular, round-shaped bacteria (Fig. 6D, upper panels, arrow). While these cells expressed IBA1 on their surface, intracellular IBA1 co-localized to the bacteria. The concentration of extracellular antigen in these regions (Fig. 6D, upper panels, arrowhead) may have been caused by successful degradation and externalization of degraded bacteria.

Contrarily, in the lung parenchyma, IBA1-positive macrophages harboring much larger numbers of intracellular bacteria were identified. IBA1 expression in these cells was mainly intracellular and co-localized with the majority of intracellular bacteria (Fig. 6D, lower panels).

Our data show that in pleura and BALT, only solitary bacteria inside macrophages were present. *O. tsutsugamushi* antigen accumulated in the extracellular space in these areas, possibly after exocytosis of degraded bacterial remnants. This bacterial degradation was likely mediated by macrophages. Infected macrophages harboring much larger numbers of intact intracellular bacteria, in contrast, were found in the lung parenchyma. In these cells, IBA1 expression was focused around the intracellular bacteria rather than on the cell membrane. Importantly, no bacteria were found in CD31-positive endothelial cells despite a close spatial relationship.

### Organ-specific kinetics of macrophage activation

**Figure 7:**
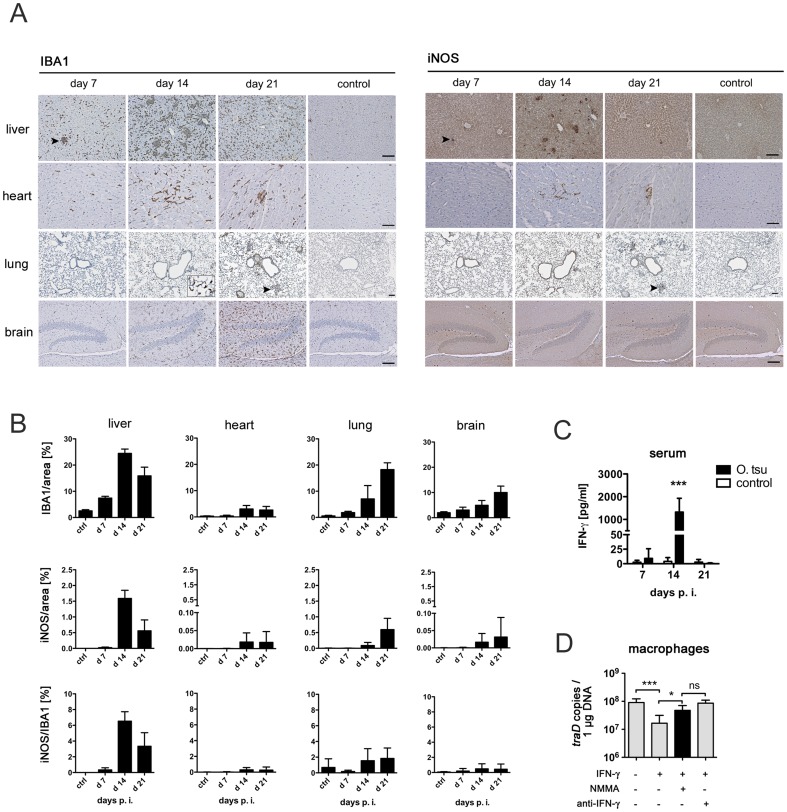
Patterns of macrophage infiltration and activation: contributions of the IFN-γ-/iNOS-axis to growth control of *O. tsutsugamushi in vitro* and *in vivo*. Balb/c mice were infected with 5×10^3^ *sfu* of *O. tsutsugamushi* via the hind footpad, or mock-infected. A, Serial sections from liver, heart, lung and brain obtained at day 7, 14 and 21 p.i. and mock controls were analyzed by IHC for macrophages (IBA1) and iNOS expression. Arrowheads point to areas of IBA1-/iNOS-double positive macrophage infiltrates. Scale bars: 50 µm. B, IBA1- and iNOS-positive areas were quantified and expressed as percentage of the entire analyzed area (upper and middle row) or as iNOS/IBA1 ratio (lower row). Data are depicted as mean ± SD (n = 10–14 areas from 2 animals). C, IFN-γ was measured in serum samples taken on d 7, 14 and 21 p.i. Results are combined data from two independent experiments (n = 5–7; mean ± SD). ***, p<0.001 by two-way ANOVA with Bonferroni's post correction. D, In 24-well plates, 2×10^5^ macrophages were infected with purified *O. tsutsugamushi* and grown in the presence of absence of recombinant IFN-γ (100 IU), the iNOS inhibitor N-monomethylarginine (NMMA, 1 mM) or the anti-IFN-γ mAb XMG1.2 (1 µg/ml). After 3 days, DNA was extracted, and the bacterial load was quantified by *traD* qPCR. IFN-γ reduced the growth of *O. tsutsugamushi* significantly, while anti-IFN-γ and NMMA reversed the IFN-γ-induced growth reduction. Results are combined from two independent experiments (mean ± SD); *, p<0.05; ***, p<0.001; ns, not significant by one-way ANOVA with Bonferroni's post correction.

Lung, heart, liver and CNS are commonly involved in the clinical symptomatology of scrub typhus. In our model, they showed differences in *O. tsutsugamushi* organ loads and clearance dynamics (Fig. 2D). We wanted to know whether these differences relate to different kinetics of macrophage appearance and activation. IHC for IBA1 and iNOS was thus performed on tissue samples collected at day 7, 14 and 21 p.i. from footpad-infected Balb/c mice or mock-infected controls (Fig. 7A).

In serial sections of liver, heart and lung, iNOS staining co-localized with focal aggregates of IBA1-positive macrophages (Fig. 7A). In the brain, clear morphological alterations of IBA1-positive cells were present on day 14 p.i., when some cells had assumed a plumper shape and showed a lower degree of ramification (Fig. 7A). This morphology resembled amoeboid microglia, consistent with an activated state. Few iNOS-positive cells were present in the brain, but they also co-localized with IBA1 staining (Fig. 4A).

In order to compare the dynamics of macrophage activation, the relative tissue contents of iNOS- and IBA1-positive cells were quantified (Fig. 7B). Of the four organs analyzed, the macrophage response was fastest and strongest in the liver. Here, a 3-fold increase of macrophage content was present at day 7 p.i. Hepatic macrophage infiltration reached a peak of 25% at day 14 p.i. and decreased thereafter. In comparison, a significant increase of cardiac macrophages set in between day 7 and 14 p.i. but remained low compared to other organs.

The macrophage response in lung and brain was protracted, with highest values at day 21 p.i.

Induction of iNOS was strongest in the liver with an iNOS/IBA1 ratio of about 6% at day 14 p.i., In heart and brain, the iNOS/IBA1 ratio remained below 0.5%. In the lung, iNOS-positive macrophages were present from day 14 p.i., with a predominance in the alveolar interstitium. By day 21 p.i., iNOS-positive macrophages in the lung were mainly found in NIFs and pleura, and to a significantly lower extent in BALT areas (Fig. 5B,C). At that time, more iNOS-positive cells per area were present, but the overall iNOS/IBA1 ratio in the lung had not further increased (Fig. 7B). Overall, brain and heart were infiltrated by a very low percentage of iNOS-positive macrophages.

These findings suggest that the strongest and most rapid macrophage response occurred in the liver, while the response was reduced or delayed in heart, lung and brain. This strong response was associated with a more efficient bacterial growth control in the liver, compared to significantly higher bacterial loads in heart and lung (Fig. 2D,E). This pattern was not observed in the brain, where a delayed infiltration of IBA1-positive cells was observed despite an efficient bacterial clearance, suggesting that other antibacterial mechanisms may become involved.

### Transient IFN-γ cytokinemia and IFN-γ/iNOS-dependent growth control

Bacterial products are able to induce the expression of iNOS in macrophages [44], [45]. As seen in our experiments, the mere presence of *O. tsutsugamushi* organisms was not sufficient for iNOS expression in all organs, e.g. in the lung. IFN-γ is the most important synergistic activator of nitric oxide (NO) production by macrophages [46] and was shown to be produced during *O. tsutsugamushi* infection in other models [47], [48]. The systemic availability of significant amounts of IFN-γ in our infection model was demonstrated by ELISA from serum samples at day 14 p.i. (Fig. 7C). To analyze whether IFN-γ is able to contribute at all to bacterial elimination via the induction of iNOS in *O. tsutsugamushi* infection, an *in vitro* approach was chosen. Infected macrophages were treated with recombinant IFN-γ in the presence or absence of the iNOS inhibitor NMMA. As shown in Fig. 7C, NMMA reversed the anti-bacterial effects of IFN-γ.

IFN-γ-dependent iNOS induction is thus an important antibacterial effector mechanism in *O. tsutsugamushi* infection. The expression of iNOS in infected tissues does not correlate with the systemic availability of IFN-γ. It is possible that IFN-γ is made available locally by contact-dependent secretion to infected host cells e.g. by T lymphocytes, thereby inducing iNOS expression.

In summary, these data highlight profound differences between macrophage infiltration and activation in different target organs during acute infection with *O. tsutsugamushi* in the mouse model. The induction of iNOS seems to be subjected to a tight temporal and spatial regulation. Although IFN-γ-induced iNOS production contributes to bacterial degradation, the serum levels of IFN-γ were not sufficient to explain the iNOS expression pattern in different target organs.

## Discussion

In this study we present the kinetic analysis of a scrub typhus mouse model in inbred BALB/c mice, using the hind footpad as inoculation site. By a novel qPCR using conserved *traD* sequences as primer targets, we provide a detailed analysis of bacterial dissemination and show that *O. tsutsugamushi* infects internal organs to variable degrees, of which the lung becomes the major target organ during systemic spreading. By immunofluorescence, we show that *O. tsutsugamushi* has a predominant macrophage rather than endothelial tropism in the lung. Moreover, we identified specific lesions in lung, liver, heart and CNS and demonstrate differences in the kinetics of macrophage responses between these important target organs.

By the novel *traD* qPCR, an about 100-fold increased sensitivity in detection of the Karp strain was obtained compared to single copy gene qPCRs. Importantly, the *traD* qPCR also detected three other strains with increased sensitivity. We furthermore showed that results from *traD* quantifications correlated with results from single copy gene assays. In our model, the new qPCR allowed the comparison of bacterial loads in small samples containing only low bacterial copy numbers. This strategy proved valuable for our defined infection model. Its use in clinical diagnostics, however, is limited at this stage, due to the unknown extent of genomic variations between wild type isolates and a low degree of cross reactions with other rickettsial pathogens.

We furthermore established and analyzed the inbred Balb/c mouse model of scrub typhus. To more closely approximate the natural transmission route, a footpad infection model was chosen. Footpad inoculation allows a combined i.d./s.c. administration [16], [17] of small volumes and thus approximates the natural inoculation route by dermal chigger bites to a higher degree than earlier studies that relied on the s.c. or i.p. infection routes [11], [13], [14], [15].

The novel *traD* qPCR was used to measure bacterial organ loads after Karp strain infection over a course of four weeks. While a recent study analyzed the dissemination of three *O. tsutsugamushi* strains by qPCR in outbred mice during the first week of infection [9], the present study is, to our knowledge, the first analysis on quantification of *O. tsutsugamushi* in an experimental infection that encompasses the entire acute phase including pathogen dissemination and clearance. As shown here, *O. tsutsugamushi* first accumulated in the regional lymph node before it spread in higher numbers to internal organs, suggesting two distinct phases of dissemination. The highest loads of *O. tsutsugamushi* were found in lung and heart tissue. With regard to the similar curves of blood and organ loads, dissemination may have occurred hematogenically, but especially the lung remained positive after completion of bacteremia. Whether this protracted course in the lung really reflects delayed bacterial clearance or ongoing replication is not certain, since we do not know to what degree dead bacteria influence qPCR results. Possibly, quantification of bacterial mRNA rather than DNA may more accurately reflect bacterial replication. It will also be interesting to know whether other infection routes such as i.p. or i.v. infection predispose for different dissemination kinetics.

In the lung, two types of IBA1-positive macrophages with distinct localizations were identified as the main host cells. *O. tsutsugamushi* was found as single coccoid bacteria in macrophages within BALT; here, IBA1 was expressed on the membrane and also co-localized to intracellular bacteria. It was shown that IBA1 is translocated from the cytosol to the membrane early during phagocytosis, where it is cross-linked to filamentous actin and becomes a significant component of the phagocytic cup [49], [50]. Our finding therefore suggests that BALT macrophages contribute to bacterial phagocytosis. Contrarily, highly infected macrophages were found in the parenchyma. IBA1 expression in these cells was mainly intracellular, but the reason for the absence of IBA1 from the cell membrane in these cells remains unclear. Our findings parallel previous reports on macrophages as important host cells in humans [5], [30].

While we found bacteria that were spatially associated with lung endothelia, no true intra-endothelial infection was recognized in our model. Endothelial infection was reported to be a hallmark of lethal human infection [30], but it is not known to what degree endothelial infection occurs during self-limiting courses of scrub typhus [5].

Since pneumonia, myocarditis, hepatitis and meningoencephalitis are important determinants of morbidity in scrub typhus, we characterized the histopathological changes in lung, heart, liver and CNS. In the lung, three different types of infiltrates were defined: perivascular BALT, parenchymal noduli and pleuritic lesions. By immunofluorescence analysis we found that mainly degraded, extracellular bacterial antigen accumulated in developing BALTs and pleural lesions already at 14 days p.i., possibly as a consequence of exocytosis of bacterial remnants. In contrast, infected cells with large numbers of intracellular bacteria were mainly identified in the parenchyma. Thus, de novo formed BALT and pleuritic infiltrates could contribute to early bacterial degradation, while solitary infected cells in the parenchyma may have escaped immunosurveillance during the first two weeks of infection. However, inflammatory noduli appeared in the parenchyma by day 21 p.i. and showed a high content of iNOS-positive macrophages. Similar parenchymal structures were shown to actively contribute to pathogen clearance in a mouse model of cytomegalovirus infection and were recently termed nodular inflammatory foci (NIFs) [43]. It is possible that NIFs form de novo as specialized compartments to fight infected cells that have escaped from immune surveillance.

The pulmonary lesions recapitulate important features of pulmonary lesions in human scrub typhus [51], [52], [53]. Subpleural, interlobular septal thickening and peribronchial infiltrates demonstrated by computer tomography [52], [54] closely resemble the morphology of our findings in murine lungs. Similar to the murine infection, pulmonary inflammation occurs rather late, being usually progressive until one week after the onset of symptoms [55].

Myocardial lesions consisted mainly of macrophages and were mainly located interstitially. Further studies on scrub typhus myocarditis will have to address which cell types or subsets are either required for pathogen clearance or are responsible for myocardial damage that may lead to myocardial infarction or myocarditis [56], [57].

In the liver, transient periportal inflammation with hepatocyte loss was found as sign of acute infection, similar to human scrub typhus hepatitis [32], [58], [59]. Interestingly, transaminase elevations in infected mice involved AST rather than ALT, thus reflecting the findings of clinical studies [34], [60]. In comparison to the lung or the heart, proliferation of *O. tsutsugamushi* was low in the liver, paralleled by early macrophage activation. While the degree of liver tissue destruction was mild in our self-limiting model of scrub typhus in BALB/c mice, the strong and early onset of the hepatic immune response suggests a protective role for the liver during scrub typhus. In humans, the strong association between underlying liver cirrhosis and fatal outcomes of scrub typhus [61] underlines that a functional hepatic immune response might be a critical determinant of favorable disease courses which deserves further study.

Central nervous symptoms in scrub typhus are common [36], [37] and have early been linked to inflammation in brain and meninges [62], [63]. In our murine model, CNS lesions appeared after the bacterial load had already declined. Leptomeningeal lesions were mainly composed of macrophages and T cells. In brain parenchyma, lesions equally consisted of macrophage/microglia and T cells. Both tight vascular cuffs and infiltrative lesions were seen, suggesting a breakdown of the blood-brain barrier. Leukocyte infiltration into the parenchyma has been shown to involve temporary residency in perivascular cuffs before the outer parenchymal basement membrane is crossed [64], [65]. Probably, the vascular cuffs and the infiltrative nodule represent two subsequent steps of leukocyte invasion into the CNS, the latter giving rise to stronger induction of iNOS.

This study also shows for the first time the kinetics of phagocyte infiltration and activation in important target organs of *O. tsutsugamushi* by histopathology. Interestingly, the maximum of IBA1-positive phagocyte invasion was observed earlier in the liver compared to heart, brain and lungs. In general, invasion of leukocytes has to be preceded by transmigration through the endothelia. The different dynamics may be linked to the differential permissiveness of fenestrated endothelia in the liver on the one hand, allowing a rapid reaction of Kupffer cells, and the tight endothelia e.g. in the brain on the other [66], [67]. It remains unexplained, however, why the macrophage reaction in the lung is delayed. Moreover, the IBA1 stain could not differentiate between tissue-resident macrophages (e.g. Kupffer cells, microglia) and monocyte-derived macrophages. Sophisticated models will be needed to differentiate the origin of macrophages and mutual contributions to organ pathology and pathogen clearance [68]. The relative content of iNOS-positive macrophages was remarkably low in heart and brain. Other effector mechanisms may be preferentially induced in order to avoid iNOS-mediated tissue damage, but further studies have to show this. We furthermore showed that IFN-γ induces an iNOS-dependent reduction of bacterial growth in macrophages *in vitro*. IFN-γ serum levels were elevated during the acute infection, but correlated poorly with iNOS expression in target organs. The measurement of IFN-γ production in the respective tissues, e.g. by mRNA expression analysis, may more accurately correlate with local iNOS expression.

In conclusion, we have analyzed an experimental mouse infection that closely approximates the natural transmission and shows a strong tropism of *O. tsutsugamushi* for lung and heart tissue. While only the immune response towards the Karp strain of *O. tsutsugamushi* was analyzed in the present study, it will be interesting to assess the effect of other, especially less pathogenic, strains. This model will be useful to understand better the immunology and pathogenesis of scrub typhus.

## Supporting Information

Figure S1 **Clinical score.** The scoring system used for measurements of clinical signs is shown.
(TIFF)

Figure S2 **Increased sensitivity of detection by** ***traD*** **qPCR.** *O. tsutsugamushi* DNA was extracted from L929 cell cultures infected with the Kato, Gilliam-like or Sido strains *O. tsutsugamushi*. Half-logarithmic dilutions were prepared. Eight to sixteen replicates of each dilution (shown on the *x*-axis) were measured for the presence or absence of *O. tsutsugamushi* by single copy 16s (right panel) or multi copy *traD* qPCR (left panel). The fraction of positive results is shown on the *y*-axis. Data were processed for probit analysis to determine the LOD95.
(TIF)

Figure S3 **Liver tissue lesions.** Serial sections of liver samples from mice footpad-infected with 5×10^3^ *sfu* of *O. tsutsugamushi* or controls were processed for Masson/Goldner Trichrome and Reticulin stains. Shown are centrilobular areas (left panels) and periportal fields (right panels). Corresponding areas of the centribular areas are marked with rectangular frames and shown in magnification (right columns). Arrowheads point to areas of reticular fiber collapse in inflamed regions. Scale Bars: 50 µm.
(TIF)

Methods S1 **This file contains supplementary methods regarding monoclonal antibody generation as well as histology/immunohistochemistry staining and quantification techniques.**
(DOC)

Table S1 **Cross reaction panel: Specificity of** ***traD*** **qPCR.** Genomic DNA of 32 freshly grown bacterial, rickettsial or fungal cultures was extracted by QiaAmp DNA Mini Kit and tested by *traD* qPCR for potential cross reactions. Negative results: −, weak positive results: (+).
(DOC)
