# Supplementary figures and images for: Dissemination of Orientia tsutsugamushi and Inflammatory Responses in a Murine Model of Scrub Typhus
Source: PLoS Negl Trop Dis. 2014 Aug 14;8(8):e3064. doi: 10.1371/journal.pntd.0003064 (PMC4133189; doi:10.1371/journal.pntd.0003064)

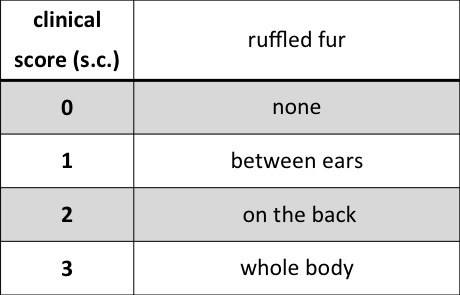

Supplement: Figure S1 — Clinical score. The scoring system used for measurements of clinical signs is shown. (TIFF) [file pntd.0003064.s001.tiff]

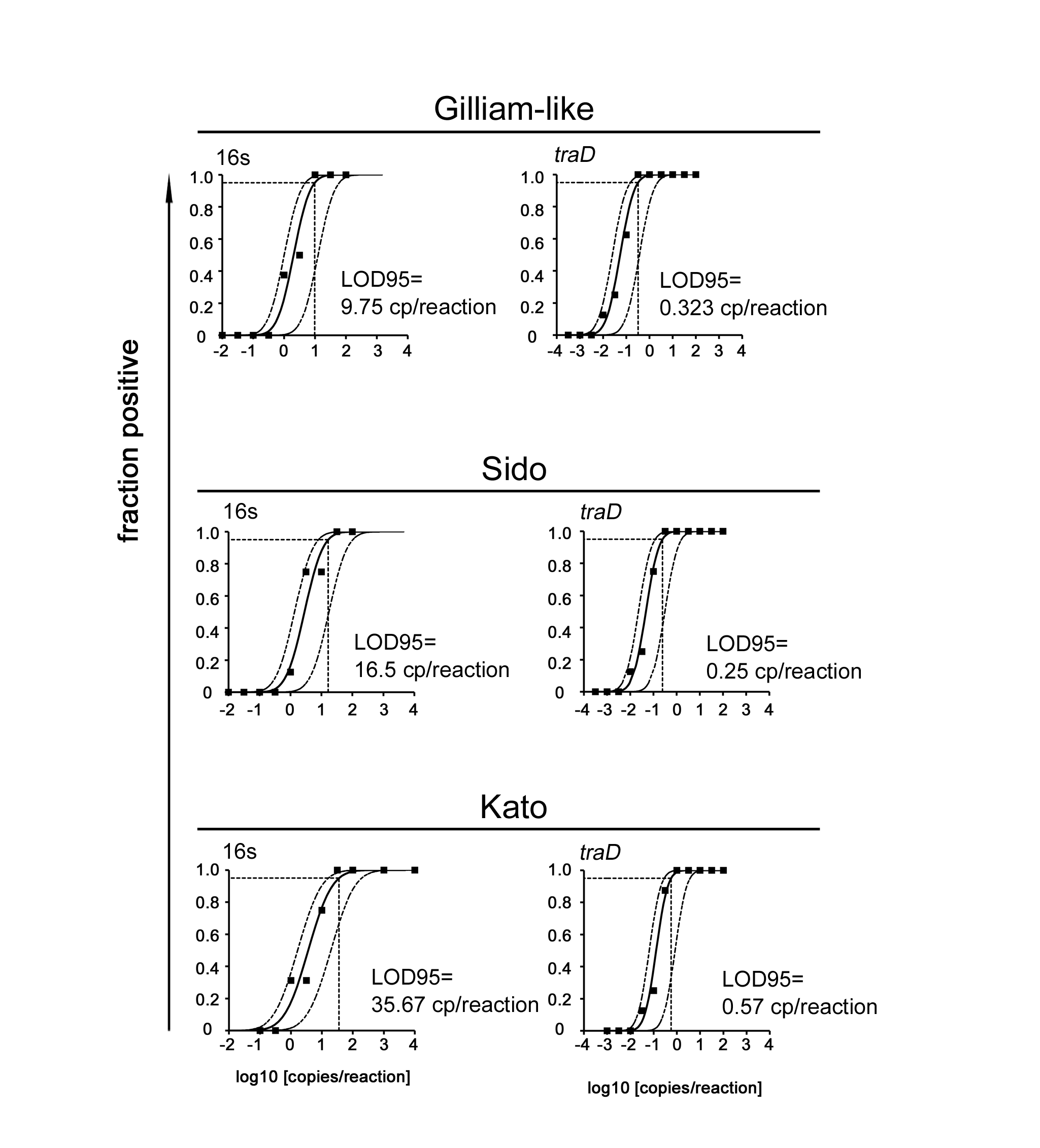

Supplement: Figure S2 — Increased sensitivity of detection by traD qPCR. O. tsutsugamushi DNA was extracted from L929 cell cultures infected with the Kato, Gilliam-like or Sido strains O. tsutsugamushi. Half-logarithmic dilutions were prepared. Eight to sixteen replicates of each dilution (shown on the x-axis) were measured for the presence or absence of O. tsutsugamushi by single copy 16s (right panel) or multi copy traD qPCR (left panel). The fraction of positive results is shown on the y-axis. Data were processed for probit analysis to determine the LOD95. (TIF) [file pntd.0003064.s002.tif]

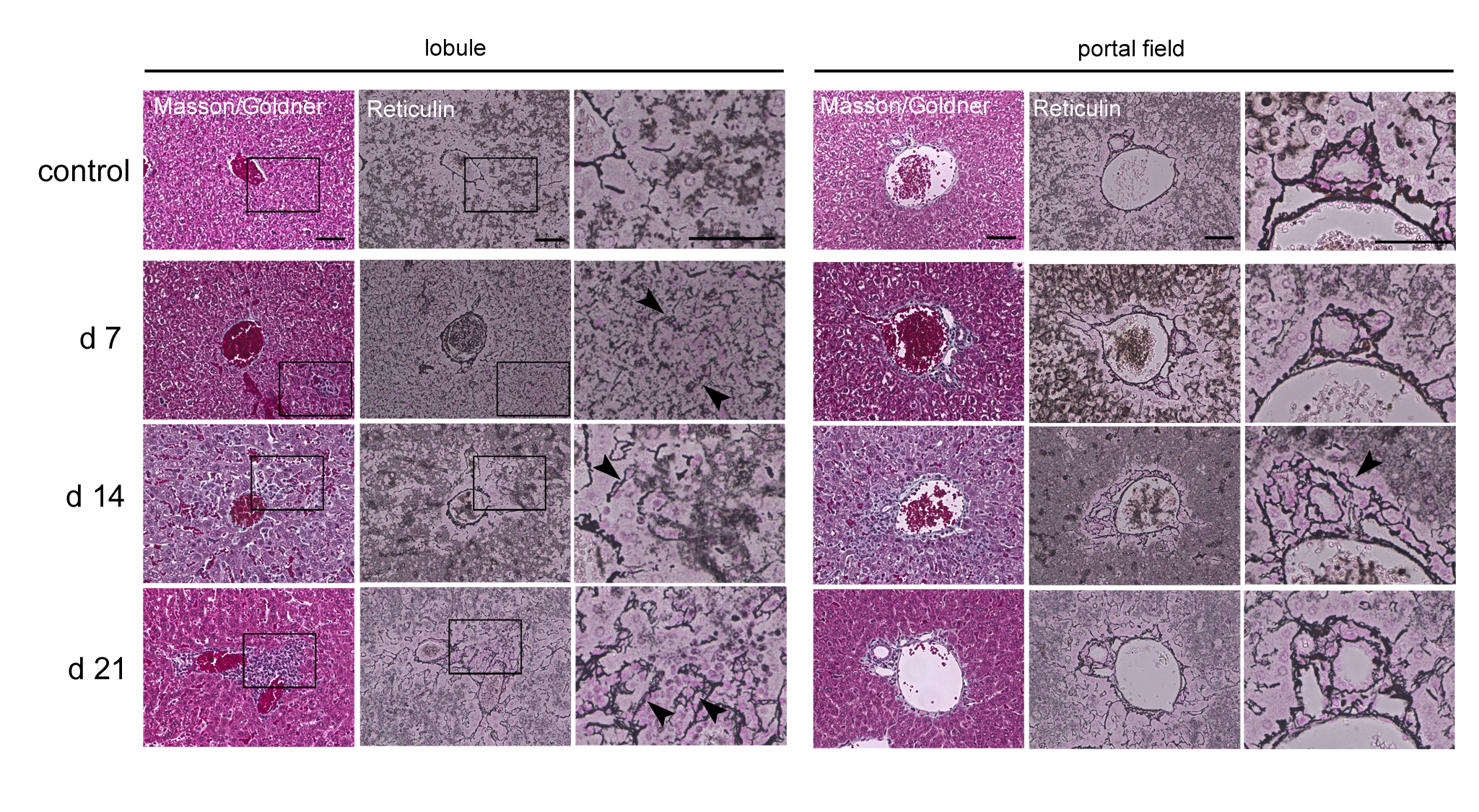

Supplement: Figure S3 — Liver tissue lesions. Serial sections of liver samples from mice footpad-infected with 5×10^3^ sfu of O. tsutsugamushi or controls were processed for Masson/Goldner Trichrome and Reticulin stains. Shown are centrilobular areas (left panels) and periportal fields (right panels). Corresponding areas of the centribular areas are marked with rectangular frames and shown in magnification (right columns). Arrowheads point to areas of reticular fiber collapse in inflamed regions. Scale Bars: 50 µm. (TIF) [file pntd.0003064.s003.tif]
