# Supplementary material for: Dissemination of Orientia tsutsugamushi and Inflammatory Responses in a Murine Model of Scrub Typhus
Source: PLoS Negl Trop Dis. 2014 Aug 14;8(8):e3064. doi: 10.1371/journal.pntd.0003064 (PMC4133189; doi:10.1371/journal.pntd.0003064)
Supplement: Methods S1 — This file contains supplementary methods regarding monoclonal antibody generation as well as histology/immunohistochemistry staining and quantification techniques. (DOC) [file pntd.0003064.s004.doc]

**Dissemination of *Orientia tsutsugamushi* and Inflammatory Responses**

**in a Murine Model of Scrub Typhus**

Christian A. Keller, Matthias Hauptmann, Julia Kolbaum, Mohammad Gharaibeh,

Melanie Neumann, Markus Glatzel, and Bernhard Fleischer

**Methods S1**

**Monoclonal antibody**

We generated a mouse monoclonal antibody (mAb) which recognizes the 56 kDa surface protein of *O. tsutsugamushi*. Briefly, female BALB/c mice immune to *O. tsutsugamushi* were boosted by intravenous (i.v.) inoculation of *O. tsutsugamushi*. After 3 days, spleen cells were fused with P3/X63-Ag8.653 myeloma cells in the presence of polyethylenglycol, and antibody-producing hybridoma cells were selected in Iscove’s medium containing hypoxanthine and azaserine. Hybridoma supernatants were screened for the presence of antibodies against *O. tsutsugamushi* by indirect immunofluorescence, and positive clones were subcloned twice. By mass spectrometry of reactive bands from a Western Blot using crude bacterial antigen, the mAb clone 2F2 was shown to be directed against the 56 kDa surface protein of *O. tsutsugamushi*.

The isotype was IgG2a- as determined with the Mouse Immunoglobulin Isotyping ELISA Kit (BD Biosciences Pharmingen, USA). An aliquot was conjugated to Alexa 488 using the Alexa Fluor 488 Protein Labeling Kit (Invitrogen, Paisley, UK).

**Histological and immunohistological stains**

Mice were anaesthetized with ketamine-xylazin. Upon cardial puncture, the lungs were perfused with sterile PBS. To obtain specimens for morphological analysis, 4% PBS-buffered formalin was instilled into the lungs intratracheally. The trachea was ligated, and organ fixation was continued in 4% formalin. Whole organs were processed to paraffin blocks, and sections of 6-8 µm were stained with hematoxylin and eosin (HE), Masson-Goldner Trichrome or Reticulin stains according to standard procedures. Sections were examined with a Nikon microscope. Imaging was performed with a BZ-9000 Keyence fluorescence microscope (Keyence, Neu-Isenburg, Germany) in brightfield mode, using a 10x magnification lens for overviews, or a 40x magnification lens for details. High contrast images were generated using full focus analysis from Z-stacks recorded with a pitch of 1.0 to 5.0 µm. For scanning of larger areas, image stitching (BZ Analyzer software, Keyence, Germany) was used.

For immunohistochemistry, sections were stained using the Ventana Benchmark XT (Ventana, Tuscon, Arizona, USA). Deparaffinized sections were boiled for 30 min in 10 mM citrate buffer (10mM sodium citrate, 0.05% Tween20, pH 6.0), for antigen retrieval. Primary antibodies were diluted in 5% goat serum (Dianova), 45 Tris-buffered saline pH 7.6 (TBS) and 0.1% Triton X-100 in antibody diluent solution (Zytomed, Berlin, Germany). Sections were incubated with primary antibody against CD3 (DAKO), B220 (eBioscience), IBA1 (WAKO), iNOS (Abcam), Ly6G (BD Biosciences) for 1 hr. Anti-rabbit or anti-rat histofine Simple Stain MAX Universal immunoperoxidase polymer (Nichirei Biosciences, Wedel, Germany) were used as secondary antibodies and detected with ultraview universal DAB detection kit (Ventana). Sections were covered (Sakura Finetek, Staufen, Germany), dried, and images were recorded as described above.

For immunofluorescence imaging, lung tissue was fixed with 4% paraformaldehyde overnight, immersed in cryopreservation medium (TissueTek O.C.T Compound, Sakura Finetek, Torrance, CA, USA), snap-frozen and sectioned with a cryostat (6-8 µm). For immunolabeling, the sections were washed in 100% ethanol for 10 min and blocked with donkey serum. Primary antibodies: goat-anti-CD31 (Santa Cruz Biotech, 1:200); rabbit-anti-IBA1 (Wako, Neuss, Germany; 1:500), diluted in TBS/donkey serum, overnight. 56 kDa *O. tsutsugamushi* surface antigen: 2F2 mAb (1:2000), diluted in PBS/0.1% Triton X-100 (Sigma, Germany)/2% FCS, 1 h. Secondary antibodies: AlexaFluor488-conjugated anti-rabbit, 1:200; AlexaFluor488-conjugated anti-goat, 1:200; AlexaFluor594-conjugated donkey-anti-mouse (1:200), 1 h in PBS/Triton/FCS (Life Technologies, Darmstadt, Germany). Nucleus counterstain: DAPI (Sigma, Germany). Sections were embedded in Fluoromount G (Southern Biotech, Birmingham, USA). Wide view immunofluorescence photographs were taken with a BZ-9000 Keyence fluorescence microscope. Details were recorded with an Olympus Confocal Microscope.

**Quantification of immunohistochemical stains**

From each organ, images from three to ten representative areas were taken with a BZ-9000 Keyence microscope. To ensure that quantification was restricted to solid organ areas, air (areas beyond organ margins, alveolar air in lung sections) and lumina of large vessels were removed from the image using the magic wand tool in Adobe Photoshop Elements 3.0. Using ImageJ software, appropriate settings for the Threshold Color tool were chosen for every organ in order to extract IBA1-/iNOS-positive or whole solid areas. The fraction of IBA1- or iNOS-positive areas per whole area was expressed in percent using the equation [IHC-positive area]/[whole area]*100.
