# Supplementary material for: Dissemination of Orientia tsutsugamushi and Inflammatory Responses in a Murine Model of Scrub Typhus
Source: PLoS Negl Trop Dis. 2014 Aug 14;8(8):e3064. doi: 10.1371/journal.pntd.0003064 (PMC4133189; doi:10.1371/journal.pntd.0003064)
Supplement: Table S1 — Cross reaction panel: Specificity of traD qPCR. Genomic DNA of 32 freshly grown bacterial, rickettsial or fungal cultures was extracted by QiaAmp DNA Mini Kit and tested by traD qPCR for potential cross reactions. Negative results: −, weak positive results: (+). (DOC) [file pntd.0003064.s005.doc]

**Table S1**

| **strain** | **result** |
| --- | --- |
| *Aeromonas hydrophila* | - |
| *Bacillus subtilis* | - |
| *Klebsiella pneumoniae* | - |
| *Listeria monocytogenes* | - |
| *Plesiomonas shigelloides* | - |
| *Proteus mirabilis OX-K* (motile) | - |
| *Pseudomonas aeruginosa* | - |
| *Salmonella paratyphi* A | - |
| *Salmonella enteritidis* D | - |
| *Salmonella typhi* D | - |
| *Shigella flexneri* | - |
| *Shigella sonnei* (smooth form) | - |
| *Staphylococcus aureus* (ß-lactamase-pos.) | - |
| *Staphylococcus epidermidis* | - |
| *Streptococcus pyogenes* A | - |
| *Streptococcus pneumoniae* | - |
| *Campylobacter jejuni* | - |
| *Candida albicans* | - |
| *Enterococcus faecalis* D | - |
| *Neisseria gonorrhoeae* | - |
| *Neisseria meningitidis* | - |
| *Vibrio parahaemolytcius* | - |
| *Vibrio cholerae* | - |
| *Yersinia enterocolitica* | - |
| *Mycobacterium avium* | - |
| *Mycobacterium intracellulare* | - |
| *Mycobacterium tuberculosis* | - |
| *Rickettsia rickettsii* | (+) |
| *Ehrlichia chaffeensis* | (+) |
| *Rickettsia conorii* | (+) |
| *Rickettsia typhi* | (+) |
| *Rickettsia africae* | - |
